# Supplementary material for: The Trichomonas vaginalis pseudocyst is a viable cell stage with a distinct transcriptome and an intact membrane
Source: PLoS Pathog. 2026 Oct 26;22(10):e1014739. doi: 10.1371/journal.ppat.1014739 (PMC13750659; doi:10.1371/journal.ppat.1014739)
Supplement: Supplementary file 3 [file ppat.1014739.s010.docx]

**Response to reviewers**

**Part I - Summary**
Please use this section to discuss strengths/weaknesses of study, novelty/significance, general execution and scholarship.

**Reviewer #1:** *In this manuscript the authors seek to demonstrate that the pseudocyst cell state is a stress-induced quiescent stage of T. vaginalis that can remain viable for days, with implications for a role in persistent infections.
Given that the pseudocyst form of T. vaginalis is a subject of interest for those seeking to better understand the role of pseudocysts in cellular events, the idea warrants further study. However, this reviewer believes that certain points in the manuscript require convincing images. All the images presented are of poor quality and do not even remotely resemble the morphology of trophozoites or pseudocysts. Furthermore, the cited literature includes articles containing erroneous concepts that the authors should address. For instance, pseudocysts are not "cyst-like," as they lack a cyst wall; the fact that they stain with WGA lectin—as reported in earlier papers by other groups—does not imply that this material is part of a cyst wall. There are numerous misinterpretations in previous articles that warrant discussion by the authors—or, at the very least, should not be cited or propagated to the scientific community, thereby perpetuating errors.*

We completely agree with the reviewer that research on the pseudocyst stage is a subject of interest in the research community, and as can occur with such topical research area, there is some dissent with some of the concepts presented. We have included more discussion of the published literature, especially those papers that the reviewer refers to, in our manuscript so as not to unduly perpetuate misconceptions.

***Reviewer #2:*** *The authors of this paper address an important and understudied question in the Trichomonas vaginalis field, whether the pseudocyst form of the parasites represents a true persistent form or a degenerative non-viable stage of the parasite. This question is of relevance as T. vaginalis prevalence does not decrease with age as is the case with other sexually transmitted infections but rather peaks in patients over 40. The existence of a true persistent/quiescent form the parasite would be significant finding in the field that could explain parasite’s unique epidemiology.
Strengths of the paper include: i) the use of multiple induction methods for pseudocyst formation (acidic stress and iron depletion), ii) a very rigorous and impressive testing of pseudocyst reconversion using cell sorting of ‘pure’ pseudocyst populations, and iii) strong transcriptional analysis of pseudocyst populations formed in response to iron depletion over time.*

We thank the reviewer for these positive comments. *The key weakness that holds back the conclusion of this paper is the inability of authors to reconvert pseudocysts back to the actively growing trophozoite state. A quiescent cell is generally thought to be on that can reactivate and resume cell division under the correct conditions. While senescence is thought to be an irreversible state of cell-cycle arrest. While the inability of the authors to reconvert pseudocysts back to the actively growing trophozoite state does not eliminate the possibility that pseudocysts are a quiescent state, it certainly does not establish the pseudocyst as such a state. The inability to reconvert pseudocysts to trophozoites argues more strongly for senescence over quiescence.
The title of the paper could equally be, “Trichomonas vaginalis pseudocysts are a senescent cell stage characterized by differentially regulated transcriptomes and intact membranes”. There is no discussion of why the author’s conclude pseudocysts are a quiescent state vs. a senescent one. Perhaps a less definitive title such as, “Trichomonas vaginalis pseudocysts are a viable cell stage characterized by differentially regulated transcriptomes and intact membranes” would be more appropriate?*

We have changed the title as suggested by the reviewer, which better represents the conclusions from the data. *Minor weakness include:
1. No testing of common morphological features pseudocyst such as flagellar internalization.*

The morphological features associated with *T. vaginalis* pseudocysts include (1) lack of externalized flagella, (2) smaller size <15 µm compared to <20 µm for trophozoites, (3) spherical shape, and (4) no motility. These features and an absence of parasite motility were observed during our experiments by light microscopy, and RNA-seq confirmed that the forms we studied were *T. vaginalis*. But further detailed testing of other common morphological features would have required access to more powerful microscopes, such as electron microscopes, which we did not have and therefore was outside the scope of this work.

*2. No analysis of Calcofluor White staining which is a commonly used method of distinguishing pseudocyst from trophozoites.*

A calcofluor white staining protocol could not be optimized in our hands, and it did not lend itself to be a useful, quantitative pseudocyst-specific dye. For this reason, we focused more on quantitative flow cytometry and transcriptomics experiments rather than microscopy-based experiments.

*3. Beri et al. 2020 shows data that parasites grown in TYM at pH 5 and 3 form viable pseudocysts that can reconvert when transferred to TYM at pH6.2. The authors of this paper show very convincing data that parasites grown at pH3.5 are not viable. Are parasites grown at pH5 also not viable? Including viability data for parasites grown at pH5 would strengthen the conclusion that pH stress is not a reliable method for pseudocyst induction.*

In our experiments, at pH 5, only 15-26% of parasites formed pseudocysts. Because the percentage of pseudocysts formed was so low, we focused on pH 3.5, and we do not have viability data for parasites grown in pH 5.

*Overall, this paper offers important contributions to the pseudocyst question in the T. vaginalis field, but the data do not fully support the conclusion that pseudocysts are a true quiescent state. If the author’s want to argue that pseudocysts are quiescent then they need to demonstrate reconversion to the trophozoite state. If the author’s want to argue that pseudocysts are viable, have intact membranes, have decreased metabolic activity compared to trophozoites, and have a distinct transcriptional profile then the data is in strong support of these conclusions.*

We thank the reviewer for this succinct conclusion from our data and completely agree. Our data indicate that pseudocysts are viable, have intact membranes, have decreased metabolic activity compared to trophozoites, and have a distinct transcriptional profile.

***Part II – Major Issues: Key Experiments Required for Acceptance***
Please use this section to detail the key new experiments or modifications of existing experiments that should be absolutely required to validate study conclusions.

Generally, there should be no more than 3 such required experiments or major modifications for a "Major Revision" recommendation. If more than 3 experiments are necessary to validate the study conclusions, then you are encouraged to recommend "Reject*".*

**Reviewer #1:**

*a non-motile pseudocyst state- For this referee it is not correct.
Although the flagella are internalized, there is intense parasite movement, as the flagella continue to beat within the vacuoles.*

The article that describes flagella beating within the vacuoles in *Tritrichomonas* foetus is by Pereira-Neves *et al*., “Tritrichomonas foetus: Budding from Multinucleated Pseudocysts”, Protist 2003, but this work does not describe motility of the pseudocyst as a whole nor does it investigate *T. vaginalis*. Our definition of “motile” refers to movement of the pseudocyst as a whole cell, and there is no literature that shows this in *T. vaginalis*, nor have we observed it in our *T. vaginalis in vitro* cultures. For this reason, and because other papers also reference the non-motility of *T. vaginalis* pseudocysts, we would like to continue to refer to them as “non-motile”.

*“or a degenerate form on its way to cell death”
-This reviewer does not agree with this statement. This point has already been discussed in several previous articles, and it is no longer considered a form of degeneration, but rather a protective mechanism against a moment of cellular stress.*

The view that pseudocysts were solely degenerate cells, versus a quiescent stress-related form, was discussed in the literature until at least 1998 (see for example Petrin D et al., “Clinical and microbiological aspects of *Trichomonas vaginalis*”, Clin Microbiol Rev 1998: “Some believe these forms to be pseudocysts, but it is more likely that they are degenerate forms of *T. vaginalis*, since they have not been reported to give rise to normal motile forms”). Later, in 2008, M. Benchimol wrote in Cell Death In Trichomonads (Programmed Cell Death in Protozoa, J.M.P. Martin, Editor): “When trichomonads are under drug treatment or nutrient depletion, cells transform into pseudocysts, which can be either a temporary/reversal form or a phase just before trichomonad cell death.” Most of the evidence for this statement comes from that group’s pioneering work on *Tr. foetus*. For example, they reported reversion of *Tr. foetus* pseudocysts (Granger et al., Parasitol Res, “Transient invagination of flagella by *Tritrichomonas foetus*.” 2000)

In our opinion, we suspect *T. vaginalis* pseudocysts *do* revert, unless they fail to encounter favorable conditions, in which case they start to die. However, we have been unable to identify favorable conditions for reversion to occur. In our view, no one has yet succeeded to revert *T. vaginalis* pseudocysts. While Benchimol et al., have reported mitosis in *T. vaginalis* pseudocysts (Pereira-Neves A, et al., “Pseudocysts in trichomonads--new insights” 2003), they have not reported reversion of *T. vaginalis* pseudocysts, and reports of *T. vaginalis* reversion by other groups are problematic for reasons stated in our paper. Also, while results can be inferred from one (tri)trichomonad species to another, it does not constitute confirmation. So we consider the ability of *T. vaginalis* pseudocysts to convert as a a still unanswered question.

To address the reviewer’s comment, we have changed the tense in the Abstract text to “…it was long debated whether the latter is a quiescent stage or a degenerate form on its way to cell death.” We also have expanded the beginning of our Discussion to cite the Benchimol 2008 view of pseudocysts as a stress response form that reverts under favorable conditions, or, failing reversion, dies.  *- Flow cytometry studies showed that pseudocysts have intact cell membranes. This is an obvious result, which has already been exhaustively demonstrated in previous articles using microscopy.*

We identified only the Beri *et al*., Front Cell Infect Microbiol 2020 paper as using flow cytometry methods to study the *T. vaginalis* pseudocyst stage. However, the authors did not use propidium iodide, and in addition we wanted to assess membrane integrity quantitatively over time: the best method for that is flow cytometry. *Tritrichomonas foetus* pseudocyst cell viability has been investigated with flow cytometry in a publication by Martínez et al., Microbiol Spectr “Prolonged survival of venereal Tritrichomonas foetus parasite in the gastrointestinal tract, bovine fecal extract, and water” 2023, and a recent publication has shown that *Tr. foetus* has a true cyst wall, unlike *T. vaginalis* (Iriarte *et al*., Nature Comms “True cyst formation underlies persistence and drug tolerance in Tritrichomonas foetus” 2026) which makes the results in these two different species challenging to compare.

*- Fluorescence-activated cell sorting studies also identified distinct sub-populations of parasites. This information had already been demonstrated in previous studies.*

The single flow cytometry study by Beri *et al.,* Front Cell Infect Microbiol 2020, did not undertake fluorescence-activated cell sorting. In addition, the subpopulations identified in the paper appear to show arbitrary gating which may have resulted in the misrepresentation of sub-populations. For these reasons we decided to undertake cell sorting experiments ourselves in addition to flow cytometry experiments. *- with four anterior flagela
Please add here that it possesses a fifth flagellum. The insertion of the undulating membrane is not sufficient for the reader to infer the presence of a fifth flagellum.*

We have inserted text to this effect as requested. *-- The third form observed in trichomonad species is immotile, smaller,
This reviewer disagrees with that statement. On the contrary, pseudocysts are larger than normal trophozoites because they undergo multiple rounds of cell division without actual cell separation, resulting in very large, multinucleated cells. Nor are they immobile; intense flagellar movement can be observed within the vacuoles. While there is no cellular displacement, there is intense movement. Previous works showed that the flagella are located in endocytic vacuoles and remain beating.*

The phenomenon of pseudocysts undergoing multiple rounds of cell division without cell separation has been reported in *Tritrichomonas foetus* but not in *T. vaginalis.* The smaller size of *T. vaginalis* pseudocysts compared to trophozoites has been reported in several publications including Dias-Lopes *et al.,* Mem Inst Oswaldo Cruz. 2017, and Afzan and Suresh 2012, Parasitol Res both referenced in our manuscript. *- Line 76: The pseudocyst form became accepted as a trichomonad response to unfavorable environmental conditions, but whether it was a protective life cycle stage or a degenerative, moribund cell generated debate, particularly for T. vaginalis [10, 13-16]. The authors are citing very old references that have already been challenged in recent articles.*

We agree that these are older references, but they provide context and background for our study which specifically investigates whether the pseudocyst stage in *T. vaginalis* is a degenerative state or viable. In addition, an article from this year Alves et al., Pharm Res “*Trichomonas vaginalis* Drug Targets and Their Role in Drug Discovery and Development” 2026, again mentions “a controversial *T. vaginalis* form widely known as pseudocysts, or more recently, cyst-like structures (CLS)”. We have added this more current reference to the manuscript (Ref 13). *Line 86: Tr. foetus.
Please, use the correct form: T. foetus. Please correct this throughout the entire text.*

When two different genera in the same text or sentence share the same initial letter (in this case “T” for *Tritrichomonas* and *Trichomonas*), standard scientific and medical style guides suggest using a multi-letter abbreviation for the genus to avoid reader confusion. This is why we have used Tr. to distinguish *Tritrichomonas* from *Trichomonas* as they are different genera. We can change this throughout the text if the reviewer prefers but this will be more confusing for readers especially since these are two quite different genera. *Line 111: Questions of what advantage suicide has for unicellular organisms, e.g., how ‘altruism’ (as understood to favor close genetic kin) could apply, hang over all such findings [31]. In this case, neither suicide nor altruism applies; it is simply a matter of survival in the face of a stressful situation.*

We have removed this sentence and the reference, as well as the senetnces in the Discussion that expand upon this.

*Line 119; A major difference between T. vaginalis growth in vivo and typical in vitro conditions is pH.
This reviewer disagrees with this statement. The primary modification arises from the fact that the culture medium is extremely artificial, failing to replicate either the presence of the vaginal microbiota or the individual's immune defense.*

We agree with the reviewer that laboratory culture media is artificial and one of the components that makes it artificial is the difference in pH. We have revised the language to be more inclusive of the other differences. *Line 120: healthy reproductive-age vagina ranges in pH from 2.8 to 4.2 (average 3.5) [33],
This reviewer disagrees with this statement.
The article cited by the authors does not reflect the true vaginal pH. A careful reading of the article in question reveals alterations in vaginal pH resulting from the dominance of lactobacilli in the environment.
I agree that the pH used in the culture medium does not exactly correspond to the normal pH. However, the average vaginal pH is 4.5 in healthy conditions.
Vaginal pH and microbicidal lactic acid when 728 lactobacilli dominate the microbiota.*

This paper (O’Hanlon et al., PLOS One 2013, Ref 33) was chosen because it classifies the pH of both healthy and reproductive age women. The vaginal pH of healthy pre-menopausal women is shown to be 2.8-4.2 in the paper; this calculation excluded patients who did not have a healthy lactobacillus-dominant vaginal microbiota. If the vaginal microbiota is not lactobacillus dominant, the proliferation of other bacterial species causes an increase in pH and vaginal dysbiosis. Menopause too increases vaginal pH to 4.5 or higher (Panda *et al*., “Vaginal pH: A marker for menopause” J Midlife Health 2014). Therefore, we prefer to keep the range of 2.8-4.2 as reported by O’Hanlon et al., because it represents the pH of healthy, reproductive age women.  *Line 126: “in media at physiological vaginal pH (pH 3–5)”
This statement contradicts the vaginal pH value presented in the previous paragraph. In this reviewer's opinion, this information is the correct one; the previous information should not be mentioned, or—if it is—the result from that other reference should be highlighted.*

Line 126 refers to the pH used by the Beri *et al*., Front Cell Infect Microbiol 2020 study. The pH range of 3-5 is within the range of healthy reproductive age vaginal pH (2.8-4.2) and post-menopausal vaginal pH (>4.5). *Additional Comments

There is incorrect information in the literature. In the published article *Demonstration and Characterization of Cyst-Like Structures in the Life Cycle of Trichomonas vaginalis* (Divya Beri et al.), the authors erroneously state that Pereira et al. (2003) found a "thick electron-dense deposition" along the cell membrane of pseudocysts—a feature supposedly completely absent in the trophozoite form. This is an absolutely incorrect claim, as that article of Pereira-Neves does not present such finding. On the contrary, no evidence of any characteristic of a cyst wall was found.*

We have removed all mention of *T. vaginalis* having a cyst wall. *Results:
Figure 1A does not show a pseudocyst. The authors should provide images at much higher magnification, showing multiple cells and using a higher-resolution microscope. In this reviewer's opinion, the arrow clearly indicates contamination rather than a pseudocyst. By observing the scale bar, it is clearly evident that it does not correspond to the size of pseudocysts, which typically measure 30 to 40 micrometers.
Even the image of the trophozoite is not of good quality. I suggest showing multiple cells with clear characteristics indicative of *Trichomonas* morphology.
In addition, In the Figure 1 legend, the authors specify "light microscope." However, what type of light microscopy? Bright-field? Phase-contrast? DIC? Furthermore, in the Materials and Methods section, the authors state that nystatin was used to prevent contamination. Now, they refer as inducing pseudocyst formation. How can the authors claim that the antibiotic induced pseudocyst formation without control experiments? The legend Only indicates the experimental procedure as “Acidic media induction of T. vaginalis pseudocysts”.*

Unfortunately, our lab is not a microscopy lab and we did not have access to high resolution microscopes at New York University where these images were taken before we left. While *Tritrichomonas foetus* pseudocysts have been shown to be 30-40 µm in length, all morphological studies of *T. vaginalis* pseudocysts have shown them to be much smaller, and even smaller than 10-20 µm *T. vaginalis* trophozoites (Kissinger et al, BMC Infectious Diseases, 2015). Of the papers that have shown image data of *T. vaginalis* pseudocysts with consistent scale bars, Beri et al (2020) showed them to be 10-15 µm, Dias-Lopes et al (2017) showed them to be 15-20 µm, and Afzan and Suresh (2012) showed them to be 10-15 µm. We have updated our legend to reflect that we used bright-field light microscopy. We are confident that our images show *T. vaginalis* pseudocysts and not “contamination” as the reviewer suggests because we extracted total RNA from the cultures, generated RNA-seq libraries, sequenced them, and all reads mapped to the *T. vaginalis* genome and no contaminating reads were identified.

*Figure 1 B. How many cells were counted? For this reviewer, the claim that 100% of a *T. vaginalis* culture forms pseudocysts within 24 hours does not align with the parameters observed in many experiments conducted by other authors.*

When cultured in pH 3.0 TYM media, all parasites changed morphologically within 24 hours as seen in Figure 1C, but were found to be dead as demonstrated by positive trypan blue staining. We saw this result consistently in all the experiments undertaken over the course of this project, but only with pseudocysts generated by the acidic media pseudocyst induction method. Since the parasites were dead, we switched tactic to another method of pseudocyst induction, iron-depletion, which generated viable pseudocysts that did not stain with trypan blue. We included the results of the acidic media induction method to highlight that this method does not induce viable pseudocysts in our hands. Under iron-depletion conditions, it took 48 hours for all parasites in our cultures to convert to pseudocysts. We note that in *Tritrichomonas* *foetus*, a smaller percentage of pseudocysts are similarly induced over a longer time period.  *Figure 1C. The authors show cells stained with Trypan blue. What does this mean for the authors? That all the cells shown were dead? If the authors indicate that the cells are not pseudocysts but rather intact cells, the legend for Figure 1A cannot be this one.*

Yes, that is correct, the cells shown in Figure 1C are dead.  *Figure 2 F is unacceptable.*

It’s unclear what is unacceptable to the reviewer about Figure 2F. To try to address any concerns we have included the complete, unedited but annotated TapeStation gel images in the Supporting Information and make clear that the three lanes shown in Figure 2F (and Figure 1D) are representative of all of the triplicate RNA extractions.

***Reviewer #2:***

*1. If the authors want to claim that pseudocysts are quiescent then they must demonstrate that pseudocysts can reconvert to trophozoites.*

We have changed the title as suggested by the reviewer to replace the word “quiescent” with “viable” and revised the text throughout the manuscript. *2. Including viability data for parasites grown at pH5 would strengthen the conclusion that pH stress is not a reliable method for pseudocyst induction.*

Please see comment in Part I above.

*3. Calcofluor white staining is typically used to differentiate pseudocysts from trophozoites. The author's should incorporate an analysis of Calcofluor white staining for their pseduocyst assays.*

Please see comment in Part I above.

***Part III – Minor Issues: Editorial and Data Presentation Modifications***
Please use this section for editorial suggestions as well as relatively minor modifications of existing data that would enhance clarity.

**Reviewer #1:** *Light microscope images have to be replaced by good images and in higher numbers.*

Unfortunately, we are not an experienced microscopy lab and we do not have access to high magnification microscopes. We would very much like to keep the images in the figures on the understanding that they show the overall staining results and thus reveal membrane integrity rather than detailed morphology of the parasites.

**Reviewer #2:** *Overall this paper is very well written.
However, it is unclear how many times the experiments in Figures 3 and 4 were performed please state this in either the methods or figure legend. Were these experiments only performed once or is this just a representative plot of multiple experiments?*

The experiment in Figure 3 was undertaken once, with two *T. vaginalis* strains each treated with six conditions simultaneously for a total of 12 *in vitro* cultures assayed. The sorting experiments in Figure 4 were done once in each of the two strains of *T. vaginalis*. The reversion experiment of iron-depletion induced pseudocysts was repeated twice and the results are shown in Figures S2 and S3. We have updated the Methods text to clarify how many times the experiments were done.
