# Supplementary material for: The Trichomonas vaginalis pseudocyst is a viable cell stage with a distinct transcriptome and an intact membrane
Source: PLoS Pathog. 2026 Oct 26;22(10):e1014739. doi: 10.1371/journal.ppat.1014739 (PMC13750659; doi:10.1371/journal.ppat.1014739)
Supplement: Supplementary file 4 [file ppat.1014739.s011.docx]

**Response to reviewers**

**Part I - Summary**

*Reviewer #1: Nothing to add.*

*Reviewer #2: The author’s data demonstrate that T. vaginalis iron deprivation induced pseudocysts are viable, have intact membranes, have decreased metabolic activity compared to trophozoites, and have a distinct transcriptional profile in comparison trophozoites.*

**Part II – Major Issues: Key Experiments Required for Acceptance**

*Reviewer #1: The authors made few changes in response to the reviewers' suggestions.
The main issue regarding the presented images was not addressed. The authors claim they lack access to a high-quality microscope; however, they should have sought access to one at another laboratory, as such a limitation is unlikely in the United States.
A publication featuring the currently presented images does not support an article containing relevant information. The authors must either provide representative images of *T. vaginalis* (trophozoites and pseudocysts) or remove them from the manuscript entirely.
This reviewer does not agree with the authors' claims.*

We have removed all images of T. vaginalis trophozoites and pseudocysts from the manuscript entirely in Fig 1 and Fig 2 and revised the legends and text.

*Reviewer #2: The authors have addressed the major concern regarding the premature conclusion that pseudocysts are a true quiescent state and have modified the title and text accordingly.*

***Part III – Minor Issues: Editorial and Data Presentation Modifications***
Please use this section for editorial suggestions as well as relatively minor modifications of existing data that would enhance clarity.

***Reviewer #1:*** *The issue with the images was not even accepted or modified.*

We have removed all the microscopy images in Fig 1 and Fig 2 and revised the legends and text.

***Reviewer #2:*** *Because viability of parasites was only tested for pH3.5 the authors should specify in the text that pH below 4 is not a viable method for inducing pseudocysts. This is an important finding, but since a previous paper (Beri, et al.) indicates pH5 induces pseudocyst formation and the authors also see pseudocyst formation at pH5 at rates of 14.9% and 25.9% depending on strain used it may be that acidic stress between pH4-5 is a viable method of live pseudocyst induction while pH3.5 is not. This is a minor point, but because viability was not tested for pH5 in this study and the conflicting reports in the literature (Beri, et al.) it is best to be precise.*

We have added text in the Results to clearly state this.
