# Supplementary material for: The Trichomonas vaginalis pseudocyst is a viable cell stage with a distinct transcriptome and an intact membrane
Source: PLoS Pathog. 2026 Oct 26;22(10):e1014739. doi: 10.1371/journal.ppat.1014739 (PMC13750659; doi:10.1371/journal.ppat.1014739)
Supplement: Peer review history

# Peer review: Decision Letter 0

**DOI:** 10.1371/journal.ppat.1014739.r001

**Authors:** Margaret Phillips, William Petri

15 Jul 2026

PPATHOGENS-D-26-01522

Trichomonas vaginalis pseudocysts are a quiescent cell stage characterized by differentially regulated transcriptomes and intact membranes

PLOS Pathogens

Dear Dr. Carlton,

Thank you for submitting your manuscript to PLOS Pathogens. After careful consideration, we feel that it has merit but does not fully meet PLOS Pathogens's publication criteria as it currently stands. Therefore, we invite you to submit a revised version of the manuscript that addresses the points raised during the review process.

Please submit your revised manuscript by Sep 13 2026 11:59PM. If you will need more time than this to complete your revisions, please reply to this message or contact the journal office at plospathogens@plos.org. When you're ready to submit your revision, log on to https://www.editorialmanager.com/ppathogens/ and select the 'Submissions Needing Revision' folder to locate your manuscript file.

Please include the following items when submitting your revised manuscript:

* A letter that responds to each point raised by the editor and reviewer(s). You should upload this letter as a separate file labeled 'Response to Reviewers'. This file does not need to include responses to any formatting updates and technical items listed in the 'Journal Requirements' section below.

* A marked-up copy of your manuscript that highlights changes made to the original version. You should upload this as a separate file labeled 'Revised Manuscript with Track Changes'.

* An unmarked version of your revised paper without tracked changes. You should upload this as a separate file labeled 'Manuscript'.

If you would like to make changes to your financial disclosure, competing interests statement, or data availability statement, please make these updates within the submission form at the time of resubmission. Guidelines for resubmitting your figure files are available below the reviewer comments at the end of this letter. As the corresponding author, your ORCID iD is verified in the submission system and will appear in the published article. PLOS supports the use of ORCID, and we encourage all coauthors to register for an ORCID iD and use it as well. Please encourage your coauthors to verify their ORCID iD within the submission system before final acceptance, as unverified ORCID iDs will not appear in the published article. Only the individual author can complete the verification step; PLOS staff cannot verify ORCID iDs on behalf of authors.

We look forward to receiving your revised manuscript.

Kind regards,

William A. Petri, Jr.

Academic Editor

PLOS Pathogens

Margaret Phillips

Section Editor

PLOS PathogensSumita Bhaduri-McIntosh

Editor-in-Chief

PLOS Pathogens

orcid.org/0000-0003-2946-9497

Michael Malim

Editor-in-Chief

PLOS Pathogens

orcid.org/0000-0002-7699-2064

**Additional Editor Comments (if provided):**

**Journal Requirements:**

If the reviewer comments include a recommendation to cite specific previously published works, please review and evaluate these publications to determine whether they are relevant and should be cited. There is no requirement to cite these works unless the editor has indicated otherwise.

1) Please ensure that the CRediT author contributions listed for every co-author are completed accurately and in full.

At this stage, the following Authors/Authors require contributions: Mari Shiratori, Francisco Callejas-Hernández, Jordan C Orosco, Steven S Sullivan, and Carlos Carmona-Fontaine. Please ensure that the full contributions of each author are acknowledged in the "Add/Edit/Remove Authors" section of our submission form.

The list of CRediT author contributions may be found here: https://journals.plos.org/plospathogens/s/authorship#loc-author-contributions

2) We noticed that you used the phrase 'data not shown' in the manuscript. We do not allow these references, as the PLOS data access policy requires that all data be either published with the manuscript or made available in a publicly accessible database. Please amend the supplementary material to include the referenced data or remove the references.

3) Please upload all main figures as separate Figure files in .tif or .eps format. For more information about how to convert and format your figure files please see our guidelines:

https://journals.plos.org/plospathogens/s/figures

4) Please upload a copy of Figure Fig 1-7 which you refer to in your text on pages 7, 9, 11, 13, 15, 16, and 19. Or, if the figure is no longer to be included as part of the submission please remove all reference to it within the text.

5) Please amend your detailed Financial Disclosure statement. This is published with the article. It must therefore be completed in full sentences and contain the exact wording you wish to be published.

i) State what role the funders took in the study. If the funders had no role in your study, please state: "The funders had no role in study design, data collection and analysis, decision to publish, or preparation of the manuscript."

ii) If any authors received a salary from any of your funders, please state which authors and which funders..

If you did not receive any funding for this study, please simply state: u201cThe authors received no specific funding for this work.u201d

6) We have amended your Competing Interest statement to comply with journal style. We kindly ask that you double check the statement and let us know if anything is incorrect.

7) PLOS now requires that authors provide the original uncropped and unadjusted images underlying all blot or gel results reported in a submission’s figures or Supporting Information files. This policy and the journal’s other requirements for blot/gel reporting and figure preparation are described in detail at https://journals.plos.org/plosone/s/figures#loc-blot-and-gel-reporting-requirements and https://journals.plos.org/plosone/s/figures#loc-preparing-figures-from-image-files.

When you submit your revised manuscript, please ensure that your figures adhere fully to these guidelines and provide the original underlying images for all blot or gel data reported in your submission. See the following link for instructions on providing the original image data: https://journals.plos.org/plosone/s/figures#loc-original-images-for-blots-and-gels.

In your cover letter, please note whether your blot/gel image data are in Supporting Information or posted at a public data repository, provide the repository URL if relevant, and provide specific details as to which raw blot/gel images, if any, are not available. Email us at customercare@plos.org if you have any questions.

**Reviewers' Comments:**

Reviewer's Responses to Questions

**Part I - Summary**

Please use this section to discuss strengths/weaknesses of study, novelty/significance, general execution and scholarship.

Reviewer #1: In this manuscript the authors seek to demonstrate that the pseudocyst cell state is a stress-induced quiescent stage of T. vaginalis that can remain viable for days, with implications for a role in persistent infections.

Given that the pseudocyst form of T. vaginalis is a subject of interest for those seeking to better understand the role of pseudocysts in cellular events, the idea warrants further study. However, this reviewer believes that certain points in the manuscript require convincing images. All the images presented are of poor quality and do not even remotely resemble the morphology of trophozoites or pseudocysts.Furthermore, the cited literature includes articles containing erroneous concepts that the authors should address. For instance, pseudocysts are not "cyst-like," as they lack a cyst wall; the fact that they stain with WGA lectin—as reported in earlier papers by other groups—does not imply that this material is part of a cyst wall. There are numerous misinterpretations in previous articles that warrant discussion by the authors—or, at the very least, should not be cited or propagated to the scientific community, thereby perpetuating errors.

Reviewer #2: The authors of this paper address an important and understudied question in the Trichomonas vaginalis field, whether the pseudocyst form of the parasites represents a true persistent form or a degenerative non-viable stage of the parasite. This question is of relevance as T. vaginalis prevalence does not decrease with age as is the case with other sexually transmitted infections but rather peaks in patients over 40. The existence of a true persistent/quiescent form the parasite would be significant finding in the field that could explain parasite’s unique epidemiology.

Strengths of the paper include: i) the use of multiple induction methods for pseudocyst formation (acidic stress and iron depletion), ii) a very rigorous and impressive testing of pseudocyst reconversion using cell sorting of ‘pure’ pseudocyst populations, and iii) strong transcriptional analysis of pseudocyst populations formed in response to iron depletion over time.

The key weakness that holds back the conclusion of this paper is the inability of authors to reconvert pseudocysts back to the actively growing trophozoite state. A quiescent cell is generally thought to be on that can reactivate and resume cell division under the correct conditions. While senescence is thought to be an irreversible state of cell-cycle arrest. While the inability of the authors to reconvert pseudocysts back to the actively growing trophozoite state does not eliminate the possibility that pseudocysts are a quiescent state, it certainly does not establish the pseudocyst as such a state. The inability to reconvert pseudocysts to trophozoites argues more strongly for senescence over quiescence.

The title of the paper could equally be, “Trichomonas vaginalis pseudocysts are a senescent cell stage characterized by differentially regulated transcriptomes and intact membranes”. There is no discussion of why the author’s conclude pseudocysts are a quiescent state vs. a senescent one. Perhaps a less definitive title such as, “Trichomonas vaginalis pseudocysts are a viable cell stage characterized by differentially regulated transcriptomes and intact membranes” would be more appropriate?

Minor weakness include:

1. No testing of common morphological features pseudocyst such as flagellar internalization.

2. No analysis of Calcofluor White staining which is a commonly used method of distinguishing pseudocyst from trophozoites.

3. Beri et al. 2020 shows data that parasites grown in TYM at pH 5 and 3 form viable pseudocysts that can reconvert when transferred to TYM at pH6.2. The authors of this paper show very convincing data that parasites grown at pH3.5 are not viable. Are parasites grown at pH5 also not viable? Including viability data for parasites grown at pH5 would strengthen the conclusion that pH stress is not a reliable method for pseudocyst induction.

Overall, this paper offers important contributions to the pseudocyst question in the T. vaginalis field, but the data do not fully support the conclusion that pseudocysts are a true quiescent state. If the author’s want to argue that pseudocysts are quiescent then they need to demonstrate reconversion to the trophozoite state. If the author’s want to argue that pseudocysts are viable, have intact membranes, have decreased metabolic activity compared to trophozoites, and have a distinct transcriptional profile then the data is in strong support of these conclusions.

**********

**Part II – Major Issues: Key Experiments Required for Acceptance**

Please use this section to detail the key new experiments or modifications of existing experiments that should be absolutely required to validate study conclusions.

Generally, there should be no more than 3 such required experiments or major modifications for a "Major Revision" recommendation. If more than 3 experiments are necessary to validate the study conclusions, then you are encouraged to recommend "Reject".

Reviewer #1: a non-motile pseudocyst state- For this referee it is not correct.

Although the flagella are internalized, there is intense parasite movement, as the flagella continue to beat within the vacuoles.

“or a degenerate form on its way to cell death”

-This reviewer does not agree with this statement.This point has already been discussed in several previous articles, and it is no longer considered a form of degeneration, but rather a protective mechanism against a moment of cellular stress.

- Flow cytometry studies showed that pseudocysts have intact cell membranes.

This is an obvious result, which has already been exhaustively demonstrated in previous articles using microscopy.

- Fluorescence-activated cell sorting studies also identified distinct sub-populations of parasites.

This information had already been demonstrated in previous studies.

- with four anterior flagela

Please add here that it possesses a fifth flagellum. The insertion of the undulating membrane is not sufficient for the reader to infer the presence of a fifth flagellum.

-- The third form observed in trichomonad species is immotile, smaller,

This reviewer disagrees with that statement. On the contrary, pseudocysts are larger than normal trophozoites because they undergo multiple rounds of cell division without actual cell separation, resulting in very large, multinucleated cells. Nor are they immobile; intense flagellar movement can be observed within the vacuoles. While there is no cellular displacement, there is intense movement. Previous works showed that the flagella are located in endocytic vacuoles and remain beating.

- Line 76: The pseudocyst form became accepted as a

trichomonad response to unfavorable environmental conditions, but whether it was a protective life cycle stage or a degenerative, moribund cell generated debate, particularly for T. vaginalis [10, 13-16]

The authors are citing very old references that have already been challenged in recent articles.

Line 86: Tr. foetus.

Please, use the correct form: T. foetus. Please correct this throughout the entire text.

Line 111: Questions of what advantage suicide has for

unicellular organisms, e.g., how ‘altruism’ (as understood to favor close genetic kin) could apply, hang over all such findings [31].

In this case, neither suicide nor altruism applies; it is simply a matter of survival in the face of a stressful situation.

Line 119; A major difference between T. vaginalis growth in vivo and

typical in vitro conditions is pH.

This reviewer disagrees with this statement. The primary modification arises from the fact that the culture medium is extremely artificial, failing to replicate either the presence of the vaginal microbiota or the individual's immune defense.

Line 120: healthy reproductive-age vagina ranges in pH from 2.8 to 4.2 (average 3.5) [33],

This reviewer disagrees with this statement.

The article cited by the authors does not reflect the true vaginal pH. A careful reading of the article in question reveals alterations in vaginal pH resulting from the dominance of lactobacilli in the environment.

I agree that the pH used in the culture medium does not exactly correspond to the normal pH. However, the average vaginal pH is 4.5 in healthy conditions.

Vaginal pH and microbicidal lactic acid when

728 lactobacilli dominate the microbiota.

Line 126: “in media at physiological vaginal pH (pH 3–5)”

This statement contradicts the vaginal pH value presented in the previous paragraph. In this reviewer's opinion, this information is the correct one; the previous information should not be mentioned, or—if it is—the result from that other reference should be highlighted.

Additional Comments

There is incorrect information in the literature. In the published article *Demonstration and Characterization of Cyst-Like Structures in the Life Cycle of Trichomonas vaginalis* (Divya Beri et al.), the authors erroneously state that Pereira et al. (2003) found a "thick electron-dense deposition" along the cell membrane of pseudocysts—a feature supposedly completely absent in the trophozoite form. This is an absolutely incorrect claim, as that article of Pereira-Neves does not present such finding. On the contrary, no evidence of any characteristic of a cyst wall was found.

Results:

Figure 1A does not show a pseudocyst. The authors should provide images at much higher magnification, showing multiple cells and using a higher-resolution microscope. In this reviewer's opinion, the arrow clearly indicates contamination rather than a pseudocyst. By observing the scale bar, it is clearly evident that it does not correspond to the size of pseudocysts, which typically measure 30 to 40 micrometers.

Even the image of the trophozoite is not of good quality. I suggest showing multiple cells with clear characteristics indicative of *Trichomonas* morphology.

In addition, In the Figure 1 legend, the authors specify "light microscope." However, what type of light microscopy? Bright-field? Phase-contrast? DIC? Furthermore, in the Materials and Methods section, the authors state that nystatin was used to prevent contamination. Now, they refer as inducing pseudocyst formation. How can the authors claim that the antibiotic induced pseudocyst formation without control experiments? The legend Only indicates the experimental procedure as “Acidic media induction of T. vaginalis pseudocysts”.

Figure 1 B. How many cells were counted? For this reviewer, the claim that 100% of a *T. vaginalis* culture forms pseudocysts within 24 hours does not align with the parameters observed in many experiments conducted by other authors.

Figure 1C. The authors show cells stained with Trypan blue. What does this mean for the authors? That all the cells shown were dead? If the authors indicate that the cells are not pseudocysts but rather intact cells, the legend for Figure 1A cannot be this one.

Figure 2 F is unacceptable.

Reviewer #2: 1. If the authors want to claim that pseudocysts are quiescent then they must demonstrate that pseudocysts can reconvert to trophozoites.

2. Including viability data for parasites grown at pH5 would strengthen the conclusion that pH stress is not a reliable method for pseudocyst induction.

3. Calcofluor white staining is typically used to differentiate pseudocysts from trophozoites. The author's should incorporate an analysis of Calcofluor white staining for their pseduocyst assays.

**********

**Part III – Minor Issues: Editorial and Data Presentation Modifications**

Please use this section for editorial suggestions as well as relatively minor modifications of existing data that would enhance clarity.

Reviewer #1: Light microscope images have to be replaced by good images and in higher numbers.

Reviewer #2: Overall this paper is very well written.

However, it is unclear how many times the experiments in Figures 3 and 4 were performed please state this in either the methods or figure legend. Were these experiments only performed once or is this just a representative plot of multiple experiments?

**********

PLOS authors have the option to publish the peer review history of their article (what does this mean?). If published, this will include your full peer review and any attached files.

If you choose “no”, your identity will remain anonymous but your review may still be made public.

**Do you want your identity to be public for this peer review?**  For information about this choice, including consent withdrawal, please see our Privacy Policy.

Reviewer #1: No

Reviewer #2: No

[NOTE: If reviewer comments were submitted as an attachment file, they will be attached to this email and accessible via the submission site. Please log into your account, locate the manuscript record, and check for the action link "View Attachments". If this link does not appear, there are no attachment files.]

**Figure resubmission:**

While revising your submission, we strongly recommend that you use PLOS’s NAAS tool (https://ngplosjournals.pagemajik.ai/artanalysis) to test your figure files. NAAS can convert your figure files to the TIFF file type and meet basic requirements (such as print size, resolution), or provide you with a report on issues that do not meet our requirements and that NAAS cannot fix.

After uploading your figures to PLOS’s NAAS tool - https://ngplosjournals.pagemajik.ai/artanalysis, NAAS will process the files provided and display the results in the "Uploaded Files" section of the page as the processing is complete. If the uploaded figures meet our requirements (or NAAS is able to fix the files to meet our requirements), the figure will be marked as "fixed" above. If NAAS is unable to fix the files, a red "failed" label will appear above. When NAAS has confirmed that the figure files meet our requirements, please download the file via the download option, and include these NAAS processed figure files when submitting your revised manuscript.

**Reproducibility:**

To enhance the reproducibility of your results, we recommend that authors of applicable studies deposit laboratory protocols in protocols.io, where a protocol can be assigned its own identifier (DOI) such that it can be cited independently in the future. Additionally, PLOS ONE offers an option to publish peer-reviewed clinical study protocols. Read more information on sharing protocols at https://plos.org/protocols?utm_medium=editorial-email&utm_source=authorletters&utm_campaign=protocols

# Author comment: Author response to Decision Letter 1

**DOI:** 10.1371/journal.ppat.1014739.r002

21 Aug 2026

Attachment Submitted filename: Response to reviewers.docx

# Peer review: Decision Letter 1

**DOI:** 10.1371/journal.ppat.1014739.r003

**Authors:** William Petri

22 Sep 2026

PPATHOGENS-D-26-01522R1

*Trichomonas vaginalis*  pseudocysts are a viable cell stage characterized by differentially regulated transcriptomes and intact membranes

PLOS Pathogens

Dear Dr. Carlton,

Thank you for submitting your manuscript to PLOS Pathogens. After careful consideration, we feel that it has merit but does not fully meet PLOS Pathogens's publication criteria as it currently stands. Therefore, we invite you to submit a revised version of the manuscript that addresses the points raised during the review process.

Please submit your revised manuscript by Nov 21 2026 11:59PM. If you will need more time than this to complete your revisions, please reply to this message or contact the journal office at plospathogens@plos.org. When you're ready to submit your revision, log on to https://www.editorialmanager.com/ppathogens/ and select the 'Submissions Needing Revision' folder to locate your manuscript file.

Please include the following items when submitting your revised manuscript:

* A letter that responds to each point raised by the editor and reviewer(s). You should upload this letter as a separate file labeled 'Response to Reviewers'. This file does not need to include responses to any formatting updates and technical items listed in the 'Journal Requirements' section below.

* A marked-up copy of your manuscript that highlights changes made to the original version. You should upload this as a separate file labeled 'Revised Manuscript with Track Changes'.

* An unmarked version of your revised paper without tracked changes. You should upload this as a separate file labeled 'Manuscript'.

If you would like to make changes to your financial disclosure, competing interests statement, or data availability statement, please make these updates within the submission form at the time of resubmission. Guidelines for resubmitting your figure files are available below the reviewer comments at the end of this letter.

As the corresponding author, your ORCID iD is verified in the submission system and will appear in the published article. PLOS supports the use of ORCID, and we encourage all coauthors to register for an ORCID iD and use it as well. Please encourage your coauthors to verify their ORCID iD within the submission system before final acceptance, as unverified ORCID iDs will not appear in the published article. Only the individual author can complete the verification step; PLOS staff cannot verify ORCID iDs on behalf of authors.

We look forward to receiving your revised manuscript.

Kind regards,

William A. Petri, Jr.

Section Editor

PLOS Pathogens

Margaret Phillips

Section Editor

PLOS Pathogens

Sumita Bhaduri-McIntosh

Editor-in-Chief

PLOS Pathogens

orcid.org/0000-0003-2946-9497

Dominique Soldati-Favre

Editor-in-Chief

PLOS Pathogens

orcid.org/0000-0003-4156-2109

**Additional Editor Comments (if provided):**

Please see the comments of review #2 who continues to request higher resolution microscopy of the parasite, and see if you can address this concern.

**Journal Requirements:**

1.We noted that you have included an author contributions statement in your manuscript file, and it appears to differ from the information we have on file in your submission form. Please remove the statement from your manuscript and instead ensure that the full contributions of each author are acknowledged in the ""Add/EditRemove Authors"" section of our submission form.

The list of CRediT author contributions may be found here: https://journals.plos.org/plospathogens/s/authorship#loc-author-contributions

2.Please amend your detailed Financial Disclosure statement. This is published with the article. It must therefore be completed in full sentences and contain the exact wording you wish to be published.

-State the initials, alongside each funding source, of each author to receive each grant.

3.Your current Financial Disclosure states, “Research reported in this publication was supported in part through the NYU IT High-Performance Computing resources, services, and staff expertise. MS was partially supported by a New York University MacCracken fellowship and National Institutes of Health T32 Training grant award GM132037. We acknowledge the Zegar Family Foundation for their generous support. The funders had no role in study design, data collection and analysis, decision to publish, or preparation of the manuscript.”.

However, your funding information on the submission form indicates that you received funding from “US National Institutes of Health [GM132037] - Mari Shiratori”. Please indicate by return email the full and correct funding information for your study and confirm the order in which funding contributions should appear. Please be sure to indicate whether the funders played any role in the study design, data collection and analysis, decision to publish, or preparation of the manuscript.

4.Please ensure that the Title in your manuscript file and the Title provided in your online submission form are the same.

5.Some material included in your submission may be copyrighted. According to PLOS’s copyright policy, authors who use figures or other material (e.g., graphics, clipart, maps) from another author or copyright holder must demonstrate or obtain permission to publish this material under the Creative Commons Attribution 4.0 International (CC BY 4.0) License used by PLOS journals. Please closely review the details of PLOS’s copyright requirements here: PLOS Licenses and Copyright. If you need to request permissions from a copyright holder, you may use PLOS's Copyright Content Permission form.

Please respond directly to this email and provide any known details concerning your material's license terms and permissions required for reuse, even if you have not yet obtained copyright permissions or are unsure of your material's copyright compatibility. Once you have responded and addressed all other outstanding technical requirements, you may resubmit your manuscript within Editorial Manager.

**Potential Copyright Issues:**

**Figure 1A and 1D:**   Please confirm whether you drew the images / clip-art within the figure panels by hand. If you did not draw the images, please provide (a) a link to the source of the images or icons and their license / terms of use; or (b) written permission from the copyright holder to publish the images or icons under our CC BY 4.0 license. Alternatively, you may replace the images with open source alternatives. See these open source resources you may use to replace images / clip-art:

- https://commons.wikimedia.org

- https://openclipart.org/

If the reviewer comments include a recommendation to cite specific previously published works, please review and evaluate these publications to determine whether they are relevant and should be cited. There is no requirement to cite these works unless the editor has indicated otherwise.

[Note: HTML markup is below. Please do not edit.]

**Reviewers' Comments:**

Reviewer's Responses to Questions

**Part I - Summary**

Please use this section to discuss strengths/weaknesses of study, novelty/significance, general execution and scholarship.

Reviewer #1: Nothing to add.

Reviewer #2: The author’s data demonstrate that T. vaginalis iron deprivation induced pseudocysts are viable, have intact membranes, have decreased metabolic activity compared to trophozoites, and have a distinct transcriptional profile in comparison trophozoites.

**********

**Part II – Major Issues: Key Experiments Required for Acceptance**

Please use this section to detail the key new experiments or modifications of existing experiments that should be absolutely required to validate study conclusions.

Generally, there should be no more than 3 such required experiments or major modifications for a "Major Revision" recommendation. If more than 3 experiments are necessary to validate the study conclusions, then you are encouraged to recommend "Reject".

Reviewer #1: The authors made few changes in response to the reviewers' suggestions.

The main issue regarding the presented images was not addressed. The authors claim they lack access to a high-quality microscope; however, they should have sought access to one at another laboratory, as such a limitation is unlikely in the United States.

A publication featuring the currently presented images does not support an article containing relevant information. The authors must either provide representative images of *T. vaginalis* (trophozoites and pseudocysts) or remove them from the manuscript entirely.

This reviewer does not agree with the authors' claims.

Reviewer #2: The authors have addressed the major concern regarding the premature conclusion that pseudocysts are a true quiescent state and have modified the title and text accordingly.

**********

**Part III – Minor Issues: Editorial and Data Presentation Modifications**

Please use this section for editorial suggestions as well as relatively minor modifications of existing data that would enhance clarity.

Reviewer #1: The issue with the images was not even accepted or modified.

Reviewer #2: Because viability of parasites was only tested for pH3.5 the authors should specify in the text that pH below 4 is not a viable method for inducing pseudocysts. This is an important finding, but since a previous paper (Beri, et al.) indicates pH5 induces pseudocyst formation and the authors also see pseudocyst formation at pH5 at rates of 14.9% and 25.9% depending on strain used it may be that acidic stress between pH4-5 is a viable method of live pseudocyst induction while pH3.5 is not. This is a minor point, but because viability was not tested for pH5 in this study and the conflicting reports in the literature (Beri, et al.) it is best to be precise.

**********

PLOS authors have the option to publish the peer review history of their article (what does this mean?). If published, this will include your full peer review and any attached files.

If you choose “no”, your identity will remain anonymous but your review may still be made public.

**Do you want your identity to be public for this peer review?**  For information about this choice, including consent withdrawal, please see our Privacy Policy.

Reviewer #1: No

Reviewer #2: No

[NOTE: If reviewer comments were submitted as an attachment file, they will be attached to this email and accessible via the submission site. Please log into your account, locate the manuscript record, and check for the action link "View Attachments". If this link does not appear, there are no attachment files.]

**Figure resubmission:**

While revising your submission, we strongly recommend that you use PLOS’s NAAS tool (https://ngplosjournals.pagemajik.ai/artanalysis) to test your figure files. NAAS can convert your figure files to the TIFF file type and meet basic requirements (such as print size, resolution), or provide you with a report on issues that do not meet our requirements and that NAAS cannot fix.

After uploading your figures to PLOS’s NAAS tool - https://ngplosjournals.pagemajik.ai/artanalysis, NAAS will process the files provided and display the results in the "Uploaded Files" section of the page as the processing is complete. If the uploaded figures meet our requirements (or NAAS is able to fix the files to meet our requirements), the figure will be marked as "fixed" above. If NAAS is unable to fix the files, a red "failed" label will appear above. When NAAS has confirmed that the figure files meet our requirements, please download the file via the download option, and include these NAAS processed figure files when submitting your revised manuscript.

**Reproducibility:**

To enhance the reproducibility of your results, we recommend that authors of applicable studies deposit laboratory protocols in protocols.io, where a protocol can be assigned its own identifier (DOI) such that it can be cited independently in the future. Additionally, PLOS ONE offers an option to publish peer-reviewed clinical study protocols. Read more information on sharing protocols at https://plos.org/protocols?utm_medium=editorial-email&utm_source=authorletters&utm_campaign=protocols

# Author comment: Author response to Decision Letter 2

**DOI:** 10.1371/journal.ppat.1014739.r004

3 Oct 2026

Attachment Submitted filename: Response2 to reviewers.docx

# Editor report: Decision Letter 2

**DOI:** 10.1371/journal.ppat.1014739.r005

**Authors:** William Petri

12 Oct 2026

Dear Professor Carlton,

We are pleased to inform that your manuscript, "The *Trichomonas vaginalis*  pseudocyst is a viable cell stage with a distinct transcriptome and an intact membrane", has been editorially accepted for publication at PLOS Pathogens.

Before your manuscript can be formally accepted and sent to production, you will need to complete our formatting changes, which you will receive by email within a week. Please note that your manuscript will not be scheduled for publication until you have made the required changes.

IMPORTANT NOTES

(1) Copyediting and Proofreading: The corresponding author will receive a typeset proof for review, to ensure errors have not been introduced during production. Please review the PDF proof of your manuscript carefully, as this is the last chance to correct any errors. Please note that major changes, or those which affect the scientific understanding of the work, will likely cause delays to the publication date of your manuscript.

(2) Appropriate Figure Files: Please remove all name and figure # text from your figure files. Please also take this time to check that your figures are of high resolution, which will improve the readbility of your figures and help expedite your manuscript's publication. Please note that figures must have been originally created at 300dpi or higher. Do not manually increase the resolution of your files. For instructions on how to properly obtain high quality images, please review our Figure Guidelines, with examples at: http://journals.plos.org/plospathogens/s/figures.

We encourage you to use PACE (http://pace.apexcovantage.com/), a figure-checking tool which helps authors ensure all figures meet PLOS requirements so that figures can publish in the highest quality. PACE is a digital diagnostic and conversion tool for figure files. It will provide information about any failed check(s) and, if able, will automatically convert the figure into an acceptable file that passes quality checks.

To use PACE, you must first register as a user. Then, login and navigate to the UPLOAD tab, where you will find detailed instructions on how to use the tool.

(3) Striking Image: Please upload a striking still image to accompany your article if one is available (you can include a new image or an existing one from within your manuscript). Should your paper be accepted, this image will be considered for our monthly issue image and may also appear on our website to feature your article. Please upload this as a separate file, selecting "striking image" as the file type upon upload. Please also include a separate "Other" file with a caption, including credits and any potential copyright information. Please do not include the caption in the main article file. If your image is from someone other than yourself, please ensure that the artist has read and agreed to the terms and conditions of the Creative Commons Attribution License at http://journals.plos.org/plospathogens/s/content-license. Please note that PLOS cannot publish copyrighted images.

(4) Press Release or Related Media: If you or your institution will be preparing press materials for this manuscript, please inform our press team in advance at plospathogens@plos.org as soon as possible. We ask that you contact us within one week to plan ahead of our fast Production schedule. If you need to know your paper's publication date for related media purposes, you must coordinate with our press team, and your manuscript will remain under a strict press embargo until the publication date and time.

(5) Update your Profile Information: Now that your manuscript has been provisionally accepted, please log into Editorial Manager and update your profile, if needed. Go to http://www.editorialmanager.com/ppathogens, log in, and click on the "Update My Information" link at the top of the page. Please update your user information to ensure an efficient production and billing process.

(6) LaTeX users only: Our staff will ask you to upload a TEX file in addition to the PDF before the paper can be sent to typesetting, so please carefully review our Latex Guidelines http://journals.plos.org/plospathogens/s/latex in the meantime.

Thank you again for supporting Open Access publishing; we are looking forward to publishing your work in PLOS Pathogens.

Best regards,

William A. Petri, Jr.

Section Editor

PLOS Pathogens

Margaret Phillips

Section Editor

PLOS Pathogens

Dominique Soldati-Favre

Editor-in-Chief

PLOS Pathogens

***********************************************************

# Editor report: Acceptance letter

**DOI:** 10.1371/journal.ppat.1014739.r006

**Authors:** William Petri

Dear Professor Carlton,

We are delighted to inform you that your manuscript, "The *Trichomonas vaginalis*  pseudocyst is a viable cell stage with a distinct transcriptome and an intact membrane," has been formally accepted for publication in PLOS Pathogens.

We have now passed your article onto the PLOS Production Department who will complete the rest of the pre-publication process. All authors will receive a confirmation email upon publication.

Thank you again for supporting open-access publishing; we are looking forward to publishing your work in PLOS Pathogens.

Best regards,

Dominique Soldati-Favre

Editor-in-Chief

PLOS Pathogens
